# Hypothermic cardiac arrest: prognostic factors for successful resuscitation before rewarming

**DOI:** 10.1186/s13049-024-01288-w

**Published:** 2024-11-14

**Authors:** Paweł Podsiadło, Konrad Mendrala, Hubert Hymczak, Ewelina Nowak, Anna Witt-Majchrzak, Wojciech Dąbrowski, Bartosz Miazgowski, Michał Dudek, Tomasz Darocha

**Affiliations:** 1 Department of Emergency Medicine, Jan Kochanowski University, al. IX Wieków Kielc 19A, Kielce, Poland; 2 Department of Anaesthesiology and Intensive Care, Medical University of Silesia, Katowice, Poland; 3 Faculty of Medicine and Health Sciences, Andrzej Frycz Modrzewski Kraków University, Kraków, Poland; 4 Institute of Health Sciences, Jan Kochanowski University, Kielce, Poland; 5 Department of Cardiac Surgery Provincial Specialist Hospital, Olsztyn, Poland; 6 Department of Anaesthesiology and Intensive Care, Medical University of Lublin, Lublin, Poland; 7 Emergency Department, University Hospital, Pomeranian Medical University, Szczecin, Poland; 8 Department of Emergency Medicine, Faculty of Health Sciences, University of Bielsko-Biała, Bielsko-Biała, Poland

**Keywords:** Accidental hypothermia, Cardiac arrest, Resuscitation, Return of spontaneous circulation

## Abstract

**Objective:**

To indicate factors predicting return of spontaneous circulation in patients with hypothermic cardiac arrest in the pre-rewarming period.

**Methods:**

A multicenter retrospective study was conducted. We included patients who had suffered cardiac arrest caused by severe accidental hypothermia with a core body temperature of ≤ 28 °C. Patients who had achieved return of spontaneous circulation before commencement of active rewarming at the hospital were compared to those who remained in cardiac arrest.

**Results:**

A total of 156 patients suffering hypothermic cardiac arrest were included in the study. In 14 of them (9%) resuscitation was successful before rewarming. Factors associated with return of spontaneous circulation were as follows: witnessed onset of cardiac arrest (*p* = 0.04); a higher core body temperature (*p* = 0.005) with a prognostic threshold of 24.6 °C; and a higher arterial oxygen partial pressure (*p* = 0.04) with a prognostic threshold of 81 mmHg. One patient after successful resuscitation sustained recurrence of cardiac arrest during rewarming.

**Conclusions:**

Patients with core body temperature < 25 °C, hypoxemia, and those who sustained unwitnessed hypothermic cardiac arrest have weak chances for successful resuscitation before rewarming. They can benefit from immediate transportation to an extracorporeal life support facility under continuous cardiopulmonary resuscitation. Effective rewarming and oxygenation during the prehospital period can increase the chances for return of spontaneous circulation. Recurrence of cardiac arrest during rewarming is uncommon.

## Introduction

Severe accidental hypothermia (i.e., a core body temperature of ≤ 28 °C) is associated with a risk of cardiac arrest [1]. This can be triggered by external stimuli or occur spontaneously [2, 3]. While factors associated with a higher risk of hypothermic cardiac arrest (HCA) have been identified [4, 5], predictors of successful prehospital resuscitation remain unknown.

Based on pathophysiology and clinical evidence, an increase in core body temperature (Tc) of a hypothermic patient with HCA raises the chances for return of spontaneous circulation (ROSC) [6]. Rewarming, especially extracorporeal rewarming, is thus the best support for resuscitation efforts [7]. However, Extracorporeal Life Support (ECLS) is not available in every hospital, and some non-medical factors can affect decisions regarding patient transportation [8]. Less-invasive rewarming methods, although effective in patients with spontaneous circulation, are linked to poorer survival rates in HCA patients [9].

On the other hand, some patients suffering HCA achieve ROSC at the prehospital or early-hospital stage. In these patients, non-ECLS rewarming can be considered. Knowledge about factors determining successful resuscitation may help clinicians to estimate the probability of ROSC and to make a decision on transportation to an ECLS facility. This could be also helpful in the prehospital triage of patients suffering severe accidental hypothermia. Those with a high risk of HCA and a low probability of ROSC before rewarming could benefit from direct transportation to an ECLS facility.

The aim of this study was to indicate factors predicting successful resuscitation of patients with HCA before commencement of rewarming.

## Methods

### Study design

We conducted a multicenter retrospective study based on patient data derived from the HELP Registry that collects the data of consecutive patient series from collaborating hospitals in Poland [4]. This study was approved by the Jan Kochanowski University Bioethical Board, consent No. 30/2024.

### Patient selection criteria

We included adult (≥ 18 y.o.) victims of accidental hypothermia with a core body temperature of ≤ 28 °C on admission to the hospital who suffered cardiac arrest before commencement of active rewarming at the hospital.

The exclusion criteria were as follows: cardiac arrest from causes other than hypothermia, known overuse of cardiovascular drugs, traumatic injury, recent brain stroke, neoplastic dissemination, cooling circumstances associated with asphyxia (e.g., avalanche burial, drowning etc.), an implanted pacemaker, and a lack of information whether the patient had achieved ROSC or not.

### Outcome

ROSC before rewarming was the primary outcome analyzed in this study. This was defined as the presence of spontaneous circulation at commencement of active rewarming in the hospital. The outcome measure was a binary variable (ROSC occurrence—or not). The rewarming modality and occurrence of HCA during rewarming among ROSC patients were secondary outcomes.

### Collected data

Age, sex, circumstances of hypothermia development (at home or outdoors), circumstances of occurrence of cardiac arrest (witnessed i.e., with vital signs at patient discovery, or unwitnessed), core body temperature on admission to the hospital, initial rhythm of cardiac arrest, duration of cardiopulmonary resuscitation (CPR), arterial blood gases (ABG) analysis on admission to the hospital (non-corrected for patient Tc), initial serum potassium and serum lactate concentrations, rewarming modality, and the occurrence of HCA during rewarming.

### Data analysis

We divided the study population into two groups, namely: patients who had achieved ROSC before commencement of rewarming in the hospital (the ROSC group) and patients who remained in cardiac arrest at commencement of rewarming (the non-ROSC group). The groups were compared by analyzing collected variables with regard to their association with ROSC occurrence.

We used the Pearson's chi-squared test to assess qualitative data, by using Yates’ correction when frequencies were below 5. The quantitative variables were analyzed with the Mann–Whitney U-test. The effect size was expressed as an Odds Ratio (OR) with a 95% Confidence Interval (CI) calculated with univariable logistic regression.

Candidate predictors for multivariable logistic regression included variables which were statistically associated with ROSC occurrence in a univariable analysis and/or seemed to be clinically important based on pathophysiology (i.e., Tc, blood gases, potassium, location of hypothermia development, initial HCA rhythm). Duration of CPR and the biochemical parameters that correlated with CPR duration were not considered as potential predictors as their values could be presumed to be the effects rather than the causes of refractoriness in HCA. For analysis, we used the Stepwise Model Builder tool that enables user-controlled selection of predictors for model building. The calibration and discrimination of developed models were assessed with the Hosmer–Lemeshow test and the Area Under the Receiver Operating Characteristic curve (AUROC). The number of independent variables in the final model was a priori limited to 2 because of a low number of ROSC events.

In addition, we estimated prognostic thresholds for parameters included in a multivariable model, by using their ROC curves with the Youden index. In order to show PaO_2_ also as temperature-corrected values, we used the calculation method described by Hansen [10].

Statistica software ver. 13.3 (Tibco Software Inc.) was used for calculations. Statistical significance was defined as *p* < 0.05.

## Results

### Patient characteristics

**Fig. 1:**
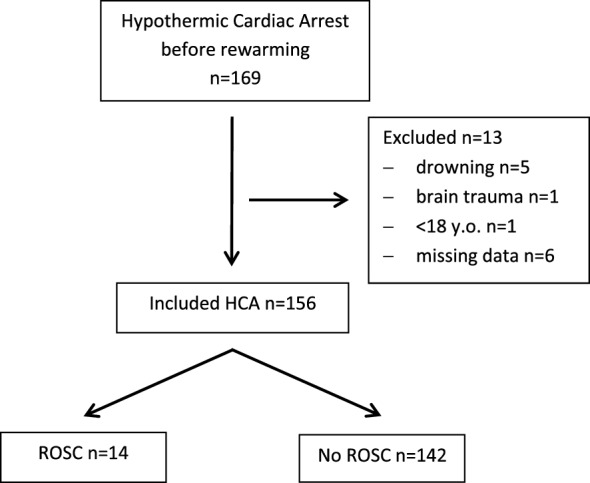
Flowchart of study population

The study population included 156 patients. Among them, 39 (25%) suffered unwitnessed HCA while 115 (75%) suffered witnessed HCA. Fourteen patients had achieved ROSC before rewarming (Fig. 1). The overall success rate in pre-rewarming CPR was 9%.

Acute (exposure) hypothermia in outdoor settings occurred in 79% of patients. The remaining 21% had become hypothermic in their homes.

The initial HCA rhythm in the entire study population was ventricular fibrillation (VF) in 65.4% patients, asystole in 27.5%, and pulseless electrical activity (PEA) in 7.1%.

The lowest Tc in a patient who achieved ROSC was 23.1 °C.

### Predictors of ROSC

The ROSC group consisted of 100% of patients suffering witnessed HCA—no patient with unwitnessed HCA had successful pre-rewarming CPR. ROSC patients had a higher Tc, higher PaO_2_, higher BE, lower lactate concentration, and a shorter duration of CPR. Details are provided in Table 1.

**Table 1:** Patients' characteristics and univariable analysis of factors associated with ROSC

Variable	Missing data	ROSC group n = 14	Non-ROSC group n = 142	*p*	Effect size OR (95% CI)
Age (years)	2%	58.5 [57–69]	55 [46–62]	0.05	1.03 (0.99–1.09)
Sex (male)	0%	11 (79%)	122 (86%)	0.7	0.66 (0.15–2.35)
Core temperature (°C)	0%	25.5 [24–27]	23.7 [22–25]	** < 0.001**	1.66 (1.21–2.27)
Circumstances of HCA (witnessed)	1%	14 (100%)	101 (72%)	**0.04**	–
Circumstances of hypothermia development (outdoors)	24%	9 (64%)	85 (81%)	0.15	0.42 (0.13–1.40)
CPR duration (min)	14%	5 [2–10]	120 [60–161]	** < 0.001**	0.79 (0.66–0.94)
Initial rhythm of HCA	2%				
Asystole		4 (33%)	38 (27%)	0.9	1.35 (0.39–4.76)
PEA		2 (17%)	9 (6%)	0.5	2.93 (0.56–15.45)
VF		6 (46%)	94 (66%)	0.1	0.44 (0.14–1.38)
Blood pH	4%	7.09 [7.03–7.18]	7.03 [6.9–7.14]	0.1	11.3 (0.40–323.6)
PaO_2_ (mmHg)	25%	91 [81–109]	65 [48–87]	**0.009**	1.01 (1.00–1.02)
PaCO_2_ (mmHg)	6%	49 [33–56]	48 [37–68]	0.6	0.99 (0.96–1.02)
BE (mmol/l)	8%	− 7.2 [− 16.5 to − 4.5]	− 16.7 [− 20 to − 12]	**0.04**	1.1 (1.01–1.19)
HCO_3_ (mmol/l)	12%	18 [11.5–25.3]	13 [10.6–16.3]	0.08	1.13 (1.03–1.24)
Serum potassium concentration (mmol/l)	5%	3.4 [2.9–3.8]	4.0 [3.2–5]	0.06	0.56 (0.31–1.02)
Serum lactate concentration (mmol/l)	8%	6.6 [3–8.6]	9.5 [6.5–12.8]	**0.004**	0.75 (0.62–0.92)
Hemoglobin level (g/dl)	12%	11.9 [10.5–13.4]	11.2 [8.8–13]	0.5	1.07 (0.88–1.30)

*p*-values in bold are statistically significant

Data presented as median [interquartile range], number (percentage); OR, odds ratio; CI, confidence interval; CPR, cardiopulmonary resuscitation; BE, base excess; HCA, hypothermic cardiac arrest; PEA, pulseless electrical activity; VF, ventricular fibrillation

Among the variables listed above, we found that BE and lactate level correlated with CPR duration. According to our assumptions described in the Methods section, these parameters were no longer analyzed as potential predictors of ROSC.

The multivariable analysis showed a significant impact of Tc and PaO_2_ on ROSC occurrence (Table 2). The AUROC of this model was 0.79 (AUC error 0.054) and the Hosmer–Lemeshow test proved not to be significant (*p* = 0.2). This reflects satisfactory discrimination and calibration, although with the limitations described below.

**Table 2:** Multivariable analysis of ROSC predictors

Variable	OR	95% CI	*p*
Core temperature	1.6	1.148–2.223	0.005
PaO_2_	1.01	1.001–1.024	0.04

OR, odds ratio; CI, confidence interval

**Fig. 2:**
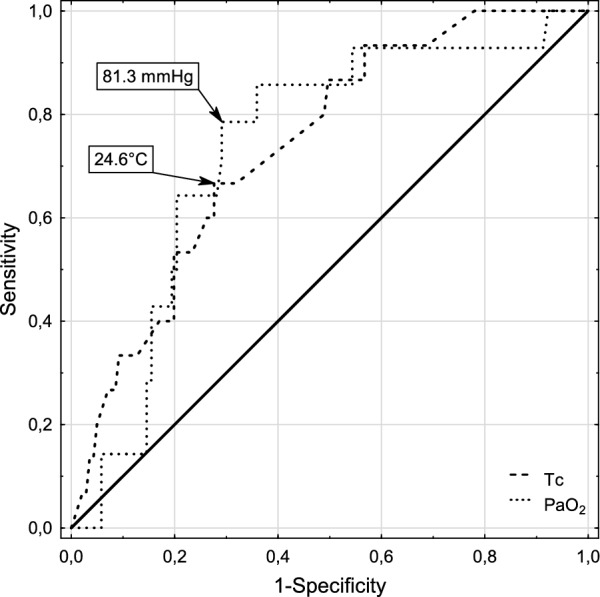
Receiver Operating Characteristic curves and cut-off values of ROSC predictors. Tc denotes core body temperature, PaO_2_—arterial oxygen partial pressure

A Tc prognostic threshold of 24.6 °C is linked to a sensitivity of 0.71 and a specificity of 0.72. A PaO_2_ threshold of 48 mmHg (corrected for patient Tc) offers a sensitivity of 0.77 and a specificity of 0.77. For the uncorrected PaO_2_ the threshold is 81 mmHg with a sensitivity of 0.77 and a specificity of 0.7 (Fig. 2).

### Post-ROSC clinical course

In nine out of 14 patients from the ROSC group, non-ECLS rewarming was started. One patient out of these 9 suffered HCA during rewarming and required a conversion to ECLS treatment while the remaining 8 retained spontaneous circulation until achieving normothermia. Five patients underwent ECLS rewarming as the first-choice treatment.

## Discussion

In this study, we showed that a witnessed onset of hypothermic cardiac arrest, a higher core body temperature and higher arterial oxygen partial pressure are associated with greater chances for successful resuscitation before rewarming. Recurrence of HCA after ROSC during non-ECLS rewarming is uncommon.

Unwitnessed HCA, although associated with relatively good chances for survival [11], is linked to a very low probability of ROSC before rewarming. Similarly, in patients with a Tc of < 24.6 °C, refractoriness of cardiac arrest is likely. Such patients should be immediately transported to an ECLS facility as their chances for ROSC without extracorporeal support are significantly reduced.

The temperature threshold of 24.6 °C distinguishing between patients with higher and with lower probability of ROSC is very close to that proposed by Mair et al. for successful defibrillation [12]. They show that converting ventricular fibrillation into a perfusing rhythm is more likely when Tc is above 24 °C. This is also consistent with the literature review by Clift and Munro-Davies where the lowest Tc in a patient with successful defibrillation is 24 °C [6]. A similar Tc threshold can be concluded from the study by Cools et al. where the lowest temperature of a patient successfully resuscitated before rewarming is 25 °C [13]. Recommendations on active rewarming of severely hypothermic patients during transportation to a hospital are not uniform. While the Wilderness Medical Society guidelines recommend active external rewarming, the International Commission for Mountain Emergency Medicine suggests avoiding prehospital rewarming of HCA patients [14, 15]. Based on our results and the cited literature, it seems likely that elevating Tc, or at least reducing its spontaneous drop during transport, can increase chances for ROSC in HCA patients.

In contrast to normothermic cardiac arrest, in severe hypothermia asystole has no negative prognostic value regarding chances for ROSC [16]. This is analogous to observations of Frei et al. and Podsiadło et al. [1, 11]. In their studies on HCA, asystole does not preclude either survival or good neurologic outcome. These clinical observations support findings of in-vitro experiments showing that HCA is mainly the effect of specific electrical disturbances in the heart, depending on temperature, without cardiac tissue damage [17].

Arterial oxygen partial pressure appeared to be significantly associated with the effectiveness of resuscitation, i.e., hypoxemia reduced chances for ROSC. Similarly, hypoxemia has been identified as a predictor of HCA occurrence [4]. This suggests that effective oxygenation may play an important role in initial management of severely hypothermic patients. Although the decreased metabolism rate in hypothermia (and thus reduced oxygen consumption) have a protective effect on brain tissue viability after cardiac arrest, blood oxygenation apparently influences the clinical course in these patients. It should be highlighted that diagnosing hypoxemia in hypothermic patients can be easier and more intuitive when temperature-corrected values are analyzed [18–21].

Recurrent cardiac arrest after ROSC is an obvious subject of clinicians’ concerns, especially in hospitals without ECLS. Mair et al. described clinical courses in hypothermic patients who sustained HCA with successful resuscitation [12]. Most of them underwent a non-ECLS rewarming without recurrence of cardiac arrest. Similarly to their findings, most of our ROSC patients retained spontaneous circulation until the end of a non-ECLS rewarming.

Lactate concentration and duration of CPR were not considered suitable in order to predict ROSC despite their statistical significance. These parameters are important predictors of survival after cardiac arrest [22–24]. However, an increase in metabolic disturbances during no-flow or low-flow conditions in tissues is not surprising, i.e., the longer the cardiac arrest time, the higher the lactate concentration [25, 26]. Therefore, sampling time seems to be crucial for values of lactate and acid–base parameters that can remain within normal range at the beginning of resuscitation efforts. An increase in CPR duration and lactate concentration is thus rather the effect of refractory cardiac arrest than its cause and not a reliable predictor of resuscitation effect [27, 28].

## Limitations

Our study suffers from several limitations. The most important weakness is its retrospective design, with no uniform protocol of patient management. This caused numerous missing data and a potential inaccuracy of results. Some patients were successfully resuscitated before arriving to the hospital and their Tc and ABG were measured after ROSC. Furthermore, this study is based on registry data that can induce a selection bias. Since our database includes only five patients with co-existing asphyxia, including them in the study population and creating a separate subgroup would not yield reliable results. Chances for pre-rewarming ROSC in this specific group of patients were thus not analyzed in our study. Finally, the relatively small sample size could reduce the reliability of results, especially a multivariable model which may be overfitted. Our results should therefore be interpreted with caution.

## Conclusions

The probability of ROSC in pre-rewarming resuscitation is higher in patients with a witnessed onset of hypothermic cardiac arrest, core body temperature > 25 °C and arterial oxygen partial pressure > 80 mmHg. Patients with a lower Tc, hypoxemia, and those who sustained unwitnessed hypothermic cardiac arrest have lower chances for ROSC before rewarming, and can thus benefit from immediate transportation to an ECLS facility. Effective rewarming and oxygenation during the prehospital period can increase chances for ROSC. Recurrence of cardiac arrest during rewarming is uncommon.

## Data Availability

The dataset analyzed in this study is available from the corresponding author on reasonable request after obtaining the consent of Bioethical Board of Jan Kochanowski University, Kielce, Poland.

## Abbreviations

ABG: Arterial blood gases
AUROC: Area under the receiver operating characteristic curve
CI: Confidence interval
CPR: Cardiopulmonary resuscitation
ECLS: Extracorporeal life support
HCA: Hypothermic cardiac arrest
OR: Odds ratio
PEA: Pulseless electrical activity
ROSC: Return of spontaneous circulation
Tc: Core temperature
VF: Ventricular fibrillation
